# A Modified Model for Quantitative Heavy Metal Source Apportionment and Pollution Pathway Identification

**DOI:** 10.3390/toxics12060382

**Published:** 2024-05-23

**Authors:** Maodi Wang, Pengyue Yu, Zhenglong Tong, Xingyuan Shao, Jianwei Peng, Yasir Hamid, Ying Huang

**Affiliations:** 1 National Engineering Laboratory of High Efficient Use on Soil and Fertilizer, College of Resources, Hunan Agricultural University, Changsha 410128, China; w1002043947@163.com (M.W.); 13203130035@163.com (P.Y.); 13203618886@stu.hunau.edu.cn (Z.T.); slock130@stu.hunau.edu.cn (X.S.); jianweipenglab@hunau.edu.cn (J.P.); 2 Ministry of Education (MOE) Key Lab of Environment, Remediation and Ecological Health, College of Environmental and Resources Science, Zhejiang University, Hangzhou 310058, China; yasirses2007@gmail.com

**Keywords:** heavy metals, source apportionment, receptor model, grouped principal components, GeoDetector

## Abstract

Current source apportionment models have successfully identified emission sources and quantified their contributions. However, when being utilized for heavy metal source apportion in soil, their accuracy needs to be improved, regarding migration patterns. Therefore, this work intended to improve the pre-existing principal component analysis and multiple linear regression with distance (PCA-MLRD) model to effectively locate pollution pathways (traffic emissions, irrigation water, atmospheric depositions, etc.) and achieve a more precise quantification. The dataset of soil heavy metals was collected from a typical area in the Chang-Zhu-Tan region, Hunan, China in 2021. The identification of the contribution of soil parent material was accomplished through enrichment factors and crustal reference elements. Meanwhile, the anthropogenic emission was identified with principal component analysis and GeoDetector. GeoDetector was used to accurately point to the pollution source from a spatial differentiation perspective. Subsequently, the pollution pathways linked to the identified sources were determined. Non-metal manufacturing factories were found to be significant anthropogenic sources of local soil contamination, mainly through rivers and atmospheric deposition. Furthermore, the influence of irrigation water on heavy metals showed a more pronounced effect within a distance of 1000 m, became weaker after that, and then gradually disappeared. This model may offer improved technical guidance for practical production and the management of soil heavy metal contamination.

## 1. Introduction

The rapid industrial and economic developments worldwide have led to significant challenges concerning heavy metals contamination [1,2]. Nearly 20% of farmlands in China exhibit varying degrees of contamination by Cd and Pb [3,4]. The first step to address the soil heavy metals pollution is the identification and regulation of contaminants sources. Currently, soil heavy metal pollution source analysis can be categorized into qualitative and quantitative approaches. Among the quantitative analyses, principal component analysis and multiple linear regression (PCA-MLR), positive principal matrix decomposition (PMF), and the absolute principal component score are commonly employed [5]. In our recent study, we introduced a distance variable to the receptor model and proposed a modified receptor model based on principal component analysis (PCA) and multiple linear regression with distance (MLRD) [6]. The model follows the fact that the spread of heavy metals from emission sources to soil diminishes as the distance increases, and the extent of this contribution can be quantified with the distance between sampling sites and the sources. This novel model efficiently identifies the precise pollution sources and quantifies their respective contributions. Previously, a number of studies have employed this model. For instance, Zeng et al. [7] used PCA-MLRD to conduct a source analysis of soil heavy metals near a multifunctional industrial park in Anhui Province, China. This approach successfully identified the possible source locations and source types, thereby reducing the subjectivity of potential source screening. However, this model still has two unsolved issues; firstly, it failed to account for the vertical migration of heavy metals, and secondly, it could not identify pollution processes related to each source.

In addressing the issue of source identification, it is worth noting that principal component analysis has been commonly employed in previous studies. However, it is important to note that the resulting explanatory elements of the factors tend to exhibit a certain degree of similarity. To cope with this issue, researchers have proposed the concept of grouped principal component analysis (GPCA), which extends the application of PCA and enables the categorization of components into distinct groups of similar characteristics, thus allowing for more precise observations [8]. Regarding matching of emission sources to obtained factors, previous researchers used typical indicators for different types of emissions (for example, Pb for traffic emissions, Cd for industrial activities, and so on), which may lead to inaccurate results due to the overly subjective judgment. Furthermore, various factories may display variability in the release of different kinds of heavy metals. For example, the production of clothing involves the utilization of raw materials with high levels of Ni and Cr [9]. Soils near chlor-alkali factories carry high levels of Hg [10], while lead smelters are associated with high levels of Pb contamination in top soils [11]. Therefore, it is necessary to make accurate judgments of potential emissions instead of identifying natural or industrial source types.

Heavy metals from pollution sources are released through sewage discharge, solid waste, and exhaust gases, which can pollute the soil through water irrigation and atmospheric deposition, as well as solid waste diffusion [12,13,14,15]. Atmospheric deposition is a global problem with serious implications for the environment and human health [16,17,18]. Previous studies have emphasized the significance of atmospheric deposition as a major contributor to heavy metals accumulation in soils [19,20]. It is well established that a reduction in atmospheric heavy metals input into agricultural soils can effectively decrease the concentration of metals in crops [21]. Researchers analyzing the sources of heavy metals in French farmland soils found that atmospheric deposition is the main input for Cu, Ni, Pb, and Zn accumulation in soils [22]. Atmospheric deposition is the main source for heavy metals accumulation in farmland in England and Wales, accounting for between 25% and 85% of the total input [23]. In recent years, developing countries have gradually begun to pay attention to the pollution problems caused by atmospheric deposition. In China, industrialization has made atmospheric deposition an intermediate pathway for the migration of heavy metals, leading to more severe heavy metal pollution in the soil [24]. It has been reported that atmospheric deposition was an important source for heavy metals input in agricultural soils in Hunan Province, which accounted for 51.24–94.74% of the total input [25,26]. However, a limited number of studies have included atmospheric depositions into receptor models. Therefore, it is necessary to determine the pollution pathways of sources contributing to heavy metals accumulation in soils, and to achieve more accurate source apportionment.

To solve the aforementioned issues, this research introduced modifications to the PCA-MLRD model through utilizing the GPCA method and GeoDetector. The study was designed to accomplish the following three tasks: (1) identification of sources and quantification of the emission contributions, (2) identifying the pollution pathways while quantifying the impacts, (3) comparing the accuracy with previously existing models.

## 2. Materials and Methods

### 2.1. Overview of the Research Area

**Figure 1:**
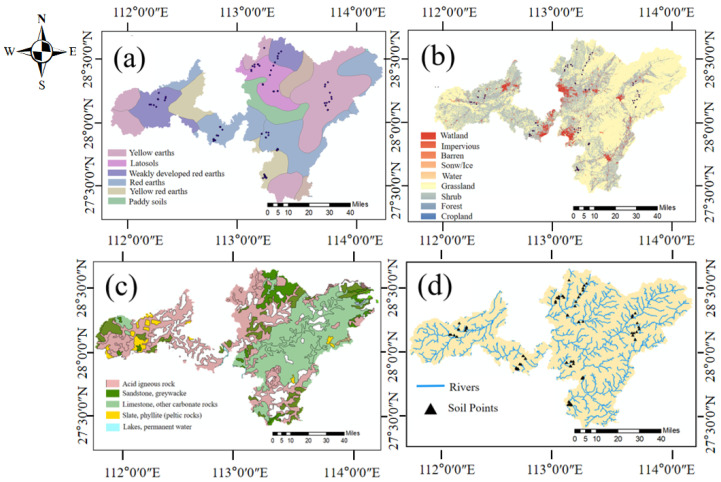
Spatial distribution map of soil types (**a**), land use (**b**), lithology (**c**), and water systems (**d**).

The study area consists of several townships belonging to 6 connected cities in Hunan Province, China, with a total area of 1333 km^2^, located at 27°24′–28°50′ N and 111°10′–114°15′ E. The study region features a subtropical monsoon climate characterized by prevailing southeast winds during summer and northeast winds in winter. The average annual temperature range is 16 °C–28 °C, with an average annual humidity of 70~80%, and an average annual precipitation estimate ranging from 1400 to about 1700 mm. The distribution maps of lithology, soil type, land use, and water are listed in Figure 1 (http://www.geodata.cn; accessed on 27 September 2022). The sampling sites were all located in paddy fields, which were dependent on the third- and fourth-grade rivers for irrigation. The study area contains six types of soils, yellow earths, latosols, weakly developed red earths, red earths, yellow red earths, and paddy soils.

### 2.2. Sampling and Analysis

**Figure 2:**
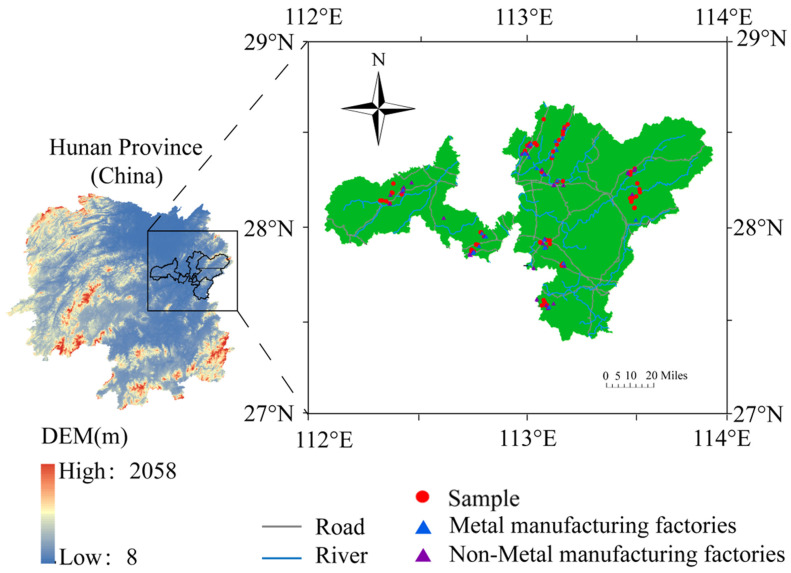
The map of the study area and locations of sampling points.

In this study, a total of 109 soil samples were collected from the cultivated layer (0–15 cm) of paddy fields. Each sampling location was recorded using a portable global positioning system (GPS), and the spatial distribution of the sampling points is illustrated in Figure 2. At the same time, a set of forty-nine dustfall cylinders (height: 30 cm; diameter: 16 cm) were placed on the roofs of residential buildings near the sampling sites to capture atmospheric depositions. The atmospheric deposition samples were collected four times throughout the year, once in each of the four seasons. During the sample collection process, the dust adhering to the bottom and inner wall of the cylinder was carefully brushed using a nylon test tube brush and thoroughly mixed with the liquid sample present in the cylinder. The resulting samples were collected in PET plastic bottles (1 L) following the addition of 10 mL of highly pure HNO_3_ and subsequently transported to the laboratory for analysis.

All soil samples were dried naturally, and the larger plants and animal residues were removed from the soil samples using plastic tweezers. Then, the soil samples were ground and passed through a 100-mesh polyethylene sieve (0.15 mm). About 0.200 g of soil was weighed and subjected to digestion on a hot plate by mixing with HNO_3_-H_2_O_2_-HF (6:3:3). Analytically pure (AR)-grade reagents were used for digestion and were obtained from Sinopharm Chemical Reagent Co., Ltd. (Shanghai, China) The atmospheric deposition samples were separated into dry and aqueous ones through filtration. Dry samples were air-dried and digested in the same way as the soil samples. The aqueous samples were extracted at 50 mL and acidified by hydrochloric acid, then digested with 2 mL HNO_3_ and 1 mL HCl at 85 °C until the samples evaporated to 20 mL. The concentrations of different elements including Cd, Ni, Cu, Zn, Cr, As, Pb, Mg, and Ca in the digested solution were detected using inductively coupled plasma mass spectrometry (ICP-MS, model NEXION 350 X; PerkinElmer, Waltham, MA, USA). To ensure the accuracy of the chemical analysis, three replicate tests, and blank reagents, as well as standard reference materials (GBW07429, China National Reference Material Research Center for Reference Materials, Beijing, China) were set up for the determination, and the recoveries of all analyses fell within the range between 90 and 110%.

### 2.3. Model Hypothesis

This study assumed that heavy metals in soil come from the parent materials and anthropogenic emissions. The anthropogenic emissions can be divided into point sources, such as factories, and non-point sources, such as traffic emissions, irrigation water, and atmospheric deposition. The main types of factories include chemical factories (cement factories, fireworks factories) that mainly emit Cd, Pb [27,28,29]; metal manufacturing factories (steel factories, electroplating factories) mainly emit Cu, As, Zn [30,31]; non-metal manufacturing factories (textile factories) mainly emit As, Cr, and Ni [32,33]. The contributions of parent materials were calculated with the enrichment factor and crustal reference element. All point sources (factories) in this area could directly emit heavy metals as sewage discharge, solid waste, and exhaust gases. In the meantime, all rivers and roads could provide channels to transfer heavy metal from the source to the sink (soil) as irrigation water and traffic emissions, where the influence becomes weaker with the increase in distance. Therefore, in the data collection, the distribution of all factories and rivers and roads was investigated. The distance refers to the shortest Euclidean distance from sampling site to factory, river, or road. In detail, traffic emissions and irrigation water are not regarded as emission sources; with the ban on leaded petrol, heavy metals from traffic emissions come mainly from the transport of industrial products and solid waste [34,35]. Therefore, to better reflect the impact of roads, traffic emissions are used as the pollutant pathway. Heavy metals in irrigation water come from industrial emissions and are, therefore, also set as a pollution pathway [36]. It has been shown that the vast majority of heavy metals in the atmosphere originate from industrial activities; they then enter the soil by means of atmospheric deposition [15,37]. Atmospheric deposition is, therefore, also studied as a pathway of industrial pollution, rather than as a source of pollution. Overall, the model includes soil-forming parent material sources and anthropogenic sources, including point and non-point sources, in its calculations.

### 2.4. Quantification of the Contribution of Soil Parent Material

Differences in lithology and soil parent material are a major cause of spatial heterogeneity of heavy metal in soils. Meanwhile, soil parent material is also thought to be the most important source of heavy metals. Therefore, the lithology of the study area was analyzed, and the contribution of soil parent material was firstly quantified with enrichment factors and crustal reference elements as the following:

(1) $$EF=\frac{{\left(\frac{\left[M\right]}{\left[C\right]}\right)}_{Soil}}{{\left(\frac{\left[M\right]}{\left[C\right]}\right)}_{Crust}}$$

(2) $${\left[M\right]}_{excessive}={\left[M\right]}_{\left(soil\right)}-{\left[C\right]}_{\left(Soil\right)}{\left(\frac{\left[M\right]}{C}\right)}_{Crust}$$

where EF: enrichment factors; [M]: the content of trace element; [C]: content of crustal reference element; ${\left(\frac{\left[M\right]}{\left[C\right]}\right)}_{Soil}$: the ratio of concentrations of trace elements in collected soil samples to crustal reference elements; ${\left(\frac{\left[M\right]}{\left[C\right]}\right)}_{Crust}$: the ratio of background values of each trace element to crustal reference elements.

### 2.5. Identification of Anthropogenic Sources and Their Potential Pathways with GPCA and GeoDetector

The application of principal component analysis (PCA) for grouping purposes sometimes encounters difficulties in identifying complex factors, particularly when dealing with a large number of elemental species, so the repetition of factor-loading elements can lead to inaccurate judgments. Therefore, in order to achieve a comprehensive division of mixed sources, the grouped principal component analysis (GPCA) method is preferred over the principal component analysis (PCA) method.

GeoDetector is a novel statistical method utilized to identify spatial heterogeneity and unveil the driving factors behind it [38,39,40]. Therefore, in this study, GeoDetector was utilized as a tool to find out the main pollution pathways of each anthropogenic source. The GeoDetector model examines the relationships between dependent variables such as heavy metal contents in soil and the explanatory variables, which include atmospheric deposition, altitude, distance to factories, and distance to roads and rivers. The altitude data corresponds to the elevation at each sampling point and was collected from the geospatial data cloud platform (http://www.gscloud.cn/; accessed on 27 September 2022). On the other hand, the distance factor represents the minimum Euclidean distance to the pollution source as well as nearby roads and rivers. It is worth noting that the explanatory variables used in GeoDetector are categorical variables. Specifically, the distance to the road, the distance to the river, the distance to the factory were discretized as classification variables, and the continuous variables should be discretized. The distances were categorized based on equal intervals of 200 m, while the elevation and atmospheric deposition were discretized into 10 categories by natural breaks. Based on the above explanatory variables and the dependent variable Y for factor detection and interaction detection, the factor detector uses the q value to measure the extent of the impact that factor (X) has on the dependent variable Y. The q values fall within the range [0, 1], with a larger q value indicating stronger explanatory power [41], and its calculation process is presented in Equation (3).

(3) $$q=1-\sum_{h=1}^{L}N_{R_{h}}\frac{\sigma_{R_{h}}^{2}}{N_{R}\sigma_{R}^{2}}$$

where $N_{R_{h}}$ and $N_{R}$ represent the grid numbers of the $R_{h}$ partition and the entire study area R, respectively. $\sigma_{R_{h}}^{2}$ and $\sigma_{R}^{2}$ are the variances of elemental heavy metal accumulation within the $R_{h}$ partition and the complete study area R, respectively.

For the interaction detector, when q(X1∩X2) > q(X1) + q(X2) for two explanatory variables, it indicates that the two factors exhibit a nonlinear connection with each other. In this study, the pollution path of the source is determined by the two explanatory variables with higher q values of nonlinear enhancement following interaction with different types of factories.

### 2.6. Quantification of the Contributions from Anthropogenic Emissions

On the basis of the PCA-MLRD model [6], the influence of pollution pathways was added to the modified model. And the modification will enhance the simulation of the pollution processes associated with emission sources. The calculation process is shown in Equation (4):

(4) $${\left[M\right]}_{excessive}=\sum_{j=1}^{p}B_{in}P_{nm}D_{mk}$$

where ${\left[M\right]}_{excessive}$ represents the cumulative content of heavy metals; $D_{mk}$ represents the distance from the source to the sampling point $;P_{nm}$ represents the main pollution pathways of source n; $B_{in}$ is the regression coefficient; n is the number of pollution sources. The mathematical transformations used in this study include logarithmic and exponential transformations for the distances matrix to find the best form of fit. In order to avoid collinearity, the stepwise regression method is used to obtain fitting parameters in multiple linear regression.

In order to maintain the accuracy of the model, samples (109) were divided into two sets: fitting sets and test sets, with the proportion of 80% (87) and 20% (22), respectively. It should be noted that the selection of fitting set and test set data was not done manually, but was carried out by using the random selection function in Excel. Moreover, four team members individually conducted the modeling separately from the start of the dataset, including the selection of crustal reference elements and model fitting. This process helps greatly reduce the subjectivity brought by artificial screening and improves the credibility of the model. Moreover, R^2^ > 0.5 was set as a basis for the test in the test set data.

### 2.7. Data Analysis

The pre-processing and statistical analysis were carried out using Microsoft Excel 2003. Graphs were created in Origin 2019 and ArcGIS 10.6. Principal component analysis and multiple linear regression for soil heavy metals were computed using SPSS 19.0 software, with the principle of eigenvalues > 1 (Kaiser criterion) to filter the principal components, while factor loadings were determined with varimax rotation [42]. ArcGIS 10.6 was used for spatial distribution mapping and shortest Euclidean distance calculation, and the inverse distance weighting (IDW) interpolation method was used for spatial analysis.

## 3. Results

### 3.1. The Statistical Analysis of Soil Heavy Metals and Quantification of Natural Sources

Descriptive statistics of soil are displayed as the maximum concentration of all the heavy metals that exceeded the standard values, with notably elevated levels of Cd and As, which exceed the standard values by 8.3 and 2.1 times, respectively (Table 1). The average concentration of heavy metals including As, Cd, Cr, Cu, Ni, Pb, Zn, Ca, and Mg in the study area were observed to be 26.47, 0.70, 87.07, 29.37, 28.41, 43.94, 133.78, 1471.28, and 4600.78 mg/kg, respectively. It was found that the mean Cd concentration exceeded the standard by 1.8 times. The ranking of heavy metals according to their coefficients of variation (CV) followed the order of Cd (57.21%), As (51.27%), Cr (42.05%), Pb (35.57%), Cu (33.99%), Ni (31.59%), and Zn (26.83%). The elemental exceedance rates were highest for Cd at 90% and smallest for Ni at 2%.

### 3.2. Analysis of Potential Pollution Sources by GPCA and Distributions of Factories

**Figure 3:**
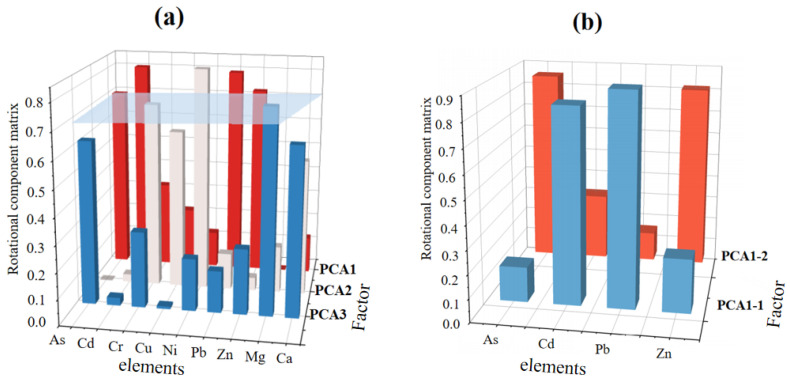
Rotated component matrices obtained by PCA (**a**) and GPCA (**b**).

PCA analysis identified three principal components, which collectively accounted for 63.85% of the total variance. Elemental loads greater than 0.7 are considered major load elements. The main interpretive elements of PCA1 include As, Cd, Pb, while those of PCA2 are Ni and Cr, and that of PCA3 is Mg (Figure 3a). However, out of the three factors mentioned above, PCA1 had four loading elements that were repeated, and the remaining two factors did not show repeated elements. Moreover, the GPCA method was used to further decompose the PCA1. The main explanatory elements of PCA1-1 are Cd (0.83), Pb (0.90), while those of PCA1-2 include As and Zn (Figure 3b). Therefore, by combining both PCA and GPCA methods, a total of four sources were identified, namely PCA1-1, PCA1-2, PCA2, PCA3.

**Figure 4:**
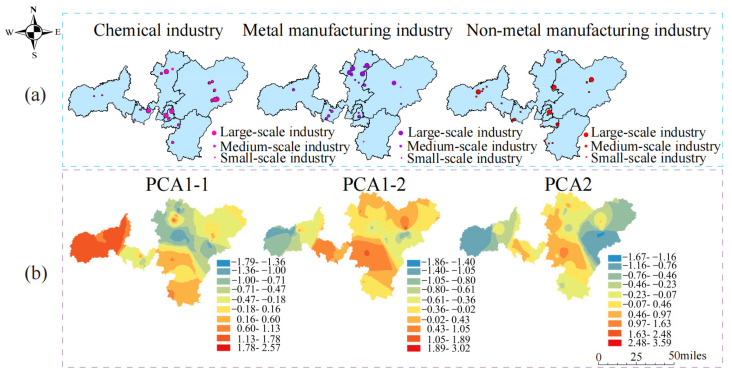
Comparison of the spatial distribution of three different types of factories (**a**) with the spatial interpolation of factor scores of the decomposed PCA model (**b**).

Figure 4 shows the spatially interpolated maps of factor scores derived from the GPCA model and spatial distribution maps of different types of factories in the studied region. The study area is predominantly characterized by the presence of cement manufacturers and fireworks factories, which were densely dispersed in the eastern and central parts. Additionally, the factor scores of PCA1-1 exhibited higher scores in the central and eastern parts of the spatial interpolation map. Meanwhile, metal manufacturing factories were predominant in the northern and central regions of the study area, aligning with higher PCA1-2 factor scores. In addition, locations with relatively high factor scores for PCA2-1 were found to have non-metal manufacturing factories, including textile factories. In general, there exists a positive relevance between higher factor scores with a denser distribution of factories and regions with large factories. The comparison revealed that the spatial interpolation of PCA1-1, PCA1-2, and PCA2 factor scores had similar patterns to the spatial distribution of chemical factories, non-metal manufacturing factories, and metal manufacturing factories in the study area.

### 3.3. Analysis of Pollution Source Pathways Based on GeoDetector

**Figure 5:**
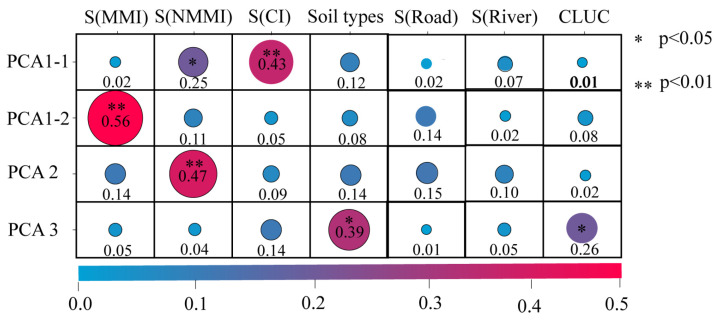
Identification results (q values) of principal component factor types using GeoDetector. Note: NMMI: non-metal manufacturing factory; CI: chemical factory; MMI: metal manufacturing factory; CLUC: classification of land use.

As shown in Figure 5, the distance to chemical plants in PCA1-1 has the highest degree of explanation (0.43); the distance to metal manufacturing plants in PCA1-2 has the highest degree of explanation (0.56) and is significantly higher than the rest of the variables; the distance to non-metal manufacturing plants in PCA2 has the highest degree of explanation (0.47); the soil type in PCA3 has the highest degree of explanation (0.47), and the land use type also has a some degree of explanation (0.26).

**Figure 6:**
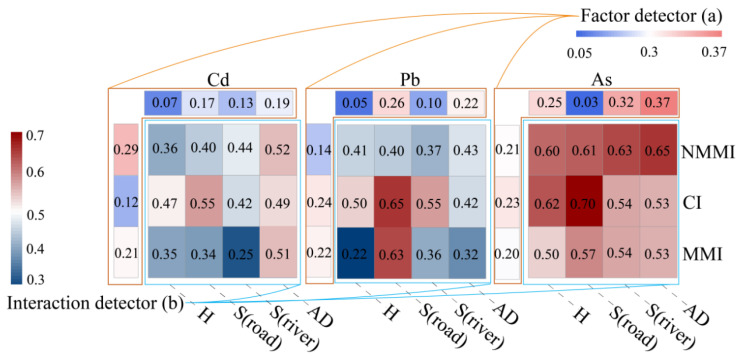
Analysis of paths by factor detectors (**a**) and interaction detectors (**b**). Note: NMMI: non-metal manufacturing factory; CI: chemical factory; MMI: metal manufacturing factory; H: altitude; AD: atmospheric deposition.

In order to identify potential contamination pathways, a combination of the factor detection and interaction detection modules of GeoDetector was employed. The identification of influencing factors was executed through GeoDetector (Figure 6a), while Figure 6b portrays the results of multiple-factor interaction detections. A large number of q values after the interaction confirmed the increment compared to the factor detection results in Figure 5. For Cd, the distance to non-metal manufacturing factories had a significant interaction with atmospheric deposition and roads, as evidenced by q values of 0.52 and 0.44, respectively. These findings suggest that Cd emissions from non-metal manufacturing factories facilitated the soil’s contamination by two pollution pathways, including atmospheric deposition and irrigation water. Similarly, the interactions between the distance of the chemical factories and the roads’ distance and atmospheric deposition indicated that traffic emissions and atmospheric deposition are the main pollution pathways for Cd emissions from chemical factories. Meanwhile, Cd pollution caused by emissions from metal manufacturing factories was affected by atmospheric deposition. In addition, the same analytical approach was employed to assess the major pollution paths of Pb and As emissions from different factories. For example, Pb pollution from non-metal manufacturing factories was influenced by atmospheric deposition, while chemical and metal manufacturing factories were primarily influenced by traffic emission and irrigation water. Concerning As pollution, non-metal manufacturing factories were linked to irrigation water and atmospheric deposition, chemical factories were associated with traffic emission, and metal manufacturing factories demonstrated a connection with traffic emission and irrigation water.

### 3.4. The Quantification of Contribution from Anthropogenic Sources

**Figure 7:**
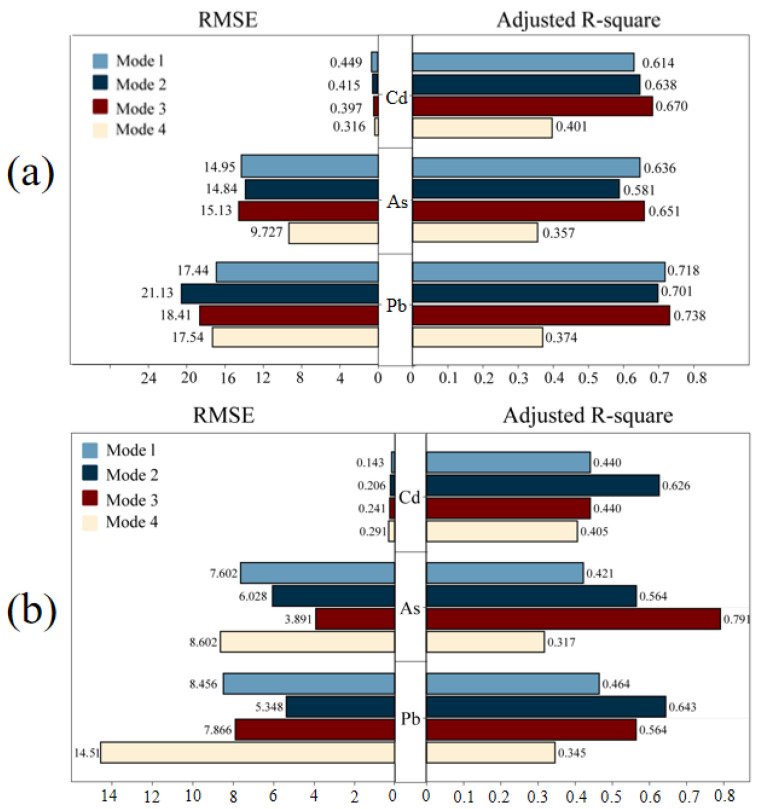
Comparison of fitting effect between four model fitting sets (**a**) and test sets (**b**).

As shown in Table 2, four models were conducted separately by four team members. In detail, Mg was used as a crustal element, and the national background values were used in model 1; Zn and Mg were used as crustal elements and the background values for different soil types were used in model 2 and model 3 (Background values for different soil types are shown in Table S1), respectively. Model 4 is the model obtained by fitting using PCA-MLRD. The conversion of the distance matrix and regression processes were both carried out separately. Equations (5)–(7) represent the regression of model 3 for Cd, As, and Pb. It can be seen that, despite the variation in contribution, the identification of sources was nearly the same. The values of R^2^ and RMSE all showed a better fitting of modified models than the original PCA-MLRD (Table 2). Based on the fitting results, it can be seen that the three anthropogenic pollution sources were finally found by the modified PCA-MLRD model, and together with the contribution of the background values, the contribution of each source was calculated separately (Figure 7). The Cd pollution was primarily caused by metal manufacturing factories (30–44%), non-metal manufacturing factories (25–35%), chemical factories (12–20%), and soil parent material (16–23%). The soil parent material sector stands out as the major contributor to Pb, reaching the largest contribution of 37–61%, while non-metal manufacturing factories had a significantly lower contribution, accounting for only 1–13%. Moreover, it was found that metal manufacturing factories were the primary source of As contamination, contributing 42–58%, while the contribution of soil parent material and non-metal manufacturing factories accounted for 14–22%, 10–17%.

(5) $$Cd=0.65\times (C_{AD}\cdot S_{road}^{\left(-\frac{2}{3}\right)})\cdot S_{NMMI}^{\left(-\frac{2}{3}\right)}+528.81\times {(C}_{AD}\cdot S_{river}^{\left(-\frac{5}{6}\right)})\cdot S_{CI}^{\left(-\frac{5}{6}\right)}+1.58\times (C_{AD}^{\left(\frac{8}{5}\right)}\cdot H)\cdot S_{MI}^{\left(-\frac{5}{8}\right)}R^{2}=0.670$$

(6) $$As=98.4\times (C_{AD}^{\left(\frac{1}{3}\right)}\cdot S_{road}^{\left(-\frac{5}{8}\right)})\cdot S_{NMMI}^{\left(-\frac{5}{8}\right)}+0.054\times (H^{\frac{5}{2}}\cdot S_{river}^{\left(-\frac{3}{4}\right)})\cdot S_{CI}^{\left(-\frac{5}{6}\right)}+14815\times (S_{road}^{\left(-\frac{3}{4}\right)}\cdot S_{river}^{\left(-\frac{3}{4}\right)})\cdot S_{MI}^{\left(-\frac{3}{4}\right)}R^{2}=0.651$$

(7) $$Pb=0.181\times (C_{AD}\cdot H)\cdot S_{NMMI}^{\left(-\frac{4}{5}\right)}+22247\times (S_{road}^{\left(-\frac{2}{3}\right)}\cdot S_{river}^{\left(-\frac{2}{3}\right)})\cdot S_{CI}^{\left(-\frac{2}{3}\right)}+3.84\times (H^{\frac{3}{2}}\cdot S_{river}^{\left(-\frac{3}{4}\right)})\cdot S_{MI}^{\left(-\frac{3}{5}\right)}R^{2}=0.738$$

where the subscript ‘NMMI’ represents the non-metal manufacturing factory; ‘CI’ means the chemical factory; ‘MI’ represented the metal manufacturing factory, and ‘AD’ represented the atmospheric deposition.

### 3.5. Effect of Pollution Pathways on Heavy Metal Accumulation in Soil

**Figure 8:**
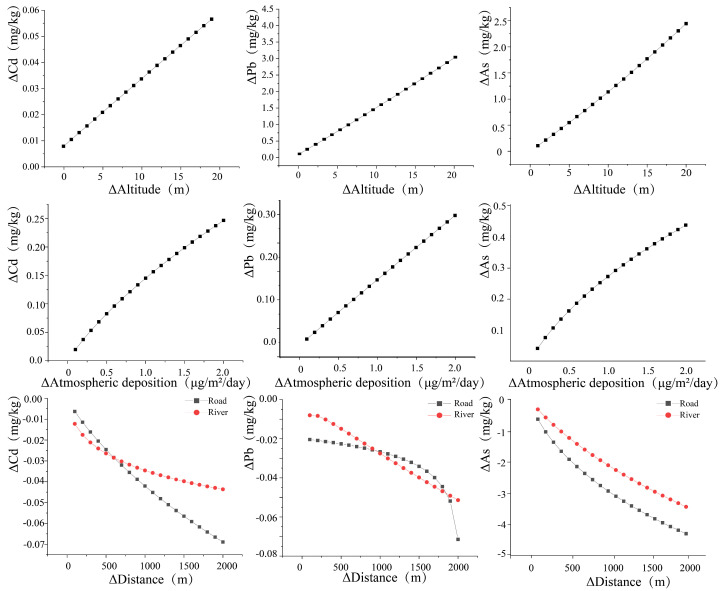
Effect of the amount of variation in different pollution pathway factors on soil heavy metals concentrations.

The effect of the pollution pathway on soil heavy metals was quantified using the model obtained above (Figure 8). It was found that two factors, including altitude and atmospheric deposition, had a positive effect on heavy metals in soil. A change in the altitude level by 1 m resulted in a significant increase in the concentration of As and Cd, with values of 0.11 mg/kg and 0.003 mg/kg, respectively. It was also observed that an increment in concentration of Cd, Pb, and As in the atmospheric deposition by 0.1 μg/m^2^/day led to elevated levels of the corresponding elements in the soil by 0.027 mg/kg, 0.016 mg/kg, and 0.04 mg/kg, respectively. In contrast, the three heavy metals displayed a nonlinear inverse relationship with both traffic emissions and irrigation water, whereas the magnitude of the effect diminished progressively as the distance increased. Specifically, for every 1000 m increase in road distance, the contents of Cd, Pb, and As in soil decreased by 0.034 mg/kg, 0.27 mg/kg, and 2.94 mg/kg, respectively. A similar trend was observed with the distance to rivers, showing decreasing heavy metals contents with an increase in distance to rivers, presenting values of 0.042 mg/kg/1000 m, 0.28 mg/kg/1000 m, and 2.11 mg/kg/1000 m, for Cd, Pb, and As, respectively. Meanwhile the amount of change in Pb increases instantly as the distance to the road increases to 1900 m. In general, the impact of roads and rivers progressively diminished and reached a state of stability as the distance extended beyond a specific threshold.

## 4. Discussion

The majority of the existing studies on pollution source analysis and identification primarily focused on the source type without identifying the actual situation of anthropogenic emissions. For example, Guan et al. (2018) employed a positive matrix factorization (PMF) model to identify the sources of soil heavy metals contamination in the Hexi Corridor region of China. In their assessment, the factors primarily explained by Cr and Ni were directly attributed to industrial sources [43]. However, the assessment technique demonstrates a significant level of subjectivity, and fails to adequately analyze the distinct categories of industrial sources. In addition, it is worth noting that other apportionment models such as principal component analysis and multiple linear regression (PCA-MLR) and Unmix also encounter similar problems [44,45]. Furthermore, a number of models can only characterize the sources into four broad categories, such as agriculture, factories, transportation, and natural sources, offering limited benefits for effectively controlling heavy metals in real practice. Therefore, our study aimed to improve the precision of specific sources, by considering the scale, type, and actual location of factories in the region. Instead of relying solely on the subjective assessment of key explanatory factors, the final analysis findings are better aligned with the actual pollution conditions in the study area.

During the source identification stage, the potential pollution sources were determined using GPCA along with the spatial distribution of the factories. The main explanatory factors of PCA1-1 included As, Cd, Pb, and Zn, and its factor scores interpolation depicted noteworthy similarities with the spatial distribution of chemical factories. Cd is a commonly used raw material in fireworks manufacturing. Pb has been identified as a distinctive component of traffic emissions in previous studies. However, with the prohibition of the usage of Pb in gasoline in China, traffic emission was no longer considered the main factor for Pb pollution [46]. The primary types of chemical factories in the study area include cement factories and fireworks factories. Previous studies have reported that As, Pb, and Cd in the raw materials of cement factories have the tendency to easily evaporate and release into the atmosphere alongside exhaust gases from cement kilns [13], while Zn is commonly used as a colorant in the production of firecrackers [31]. Further identification and comparison of pollution sources through GeoDetector found that the distance to the chemical plant had the highest degree of explanation for PCA1-1, and some studies have shown that the distance factor can reflect the degree of influence of pollution sources [47,48,49]. Therefore, the aforementioned conclusions can be combined to conclude that PCA1-1 serves as a source for chemical factories. The spatial distribution of PCA1-2 factor score interpolation showed similarities to the spatial distribution of metal manufacturing factories. The survey has revealed that the metal manufacturing factories in the study area are primarily composed of electroplating factories and steel factories. The main explanatory elements of PCA1-2 are As and Zn, which are widely used in the manufacturing of alloys, lead batteries, and semiconductor electronic devices to prepare stainless steel and corrosion-resistant alloys [44,50]. The results of GeoDetector also indicated that PCA1-2 corresponds to metal manufacturing factories. In the meantime, PCA2 mainly explained the presence of Cr and Ni, which are the main pollutants produced by garment and leather factories [32,33]. Similarly, the spatial distribution of non-metal manufacturing factories (garment factories) in the study area also showed similar characteristics to the factor scores of PCA2 in terms of the distribution of the high-value points. Therefore, PCA2 was identified as non-metal manufacturing factories. The main load elements of PCA3 are Ca and Mg and are considered to be unique elements of the soil matrix formation [51,52]. The simultaneous GeoDetector results indicate that soil type and land use type are highly explanatory for PCA3, thus identifying PCA3 as a source of soil-forming parent material.

Among the factor detector modules of the GeoDetector, it was observed that distance to roads had significant impact on As, which was further enhanced after the interaction with chemical factories. Therefore, it can be assumed that the accumulation of As in soil due to chemical factories is mainly associated with the transportation process. The results of the interaction detector module indicate that irrigation water served as the main contamination pathway for Cd and As emissions originating from non-metal manufacturing factories. The field survey revealed that the non-metal manufacturing factories were dominated by textile factories. The textile factories, being a significant contributor to wastewater, release substantial quantities of organic and inorganic pollutants into the river and atmosphere during the fabric manufacturing process [53,54,55]. Atmospheric deposition interaction analyses with non-metal manufacturing factories in the case of Cd, As and Pb showed a non-linear enhancement effect, which indicates that atmospheric deposition is the main pollution pathway from non-metal manufacturing factories in the study area. The above results on the pathways combined with the actual situation affirm the accuracy of the model in determining the potential sources of pollution. The PCA-MLRD model was employed to investigate the impact of pollution pathways on soil heavy metal concentrations. It was shown that when the distance from sampling sites to roads and rivers increased, the contents of heavy metals exhibited a significant exponential decline, which aligns with previous research [35,56]. Further comparisons between the PCA-MLRD model and the modified PCA-MLRD model revealed that the modified PCA-MLRD model had a greater accuracy, probably due to the following reasons: (1) Utilization of GPCA yielded a precise determination of types and locations of potential contamination sources, which accurately identified the actual sources of soil heavy metals contamination. (2) The original model only considered the location information of the contamination sources and relied only on distance as a representation of these contamination sources. However, the modified model added the factor of pollution pathways (traffic emissions, irrigation water, atmospheric deposition, and vertical migration) to the original model, which better simulated the contamination process of pollutants and ultimately enhanced the accuracy of results.

## 5. Conclusions

This study proposes a novel approach to heavy metal source apportionment in soil. Compared with the original PCA-MLRD, the modified model provided a more precise direction toward and basis for source identification. In addition to anthropogenic point sources, the model also accounted for non-point sources such as irrigation water, atmospheric emissions, and traffic emissions as pollution pathways. The accuracy of the model was improved, leading to more realistic results. The results indicated that metal factories had the highest contribution to Cd and As accumulations in soil, reaching 30–44% and 42–58%, respectively, and the highest contribution to Pb came from soil parent material, reaching 37–61%. The extent of the influence of pathway factors on soil heavy metals was also quantified. In terms of application scenarios, the model is more useful for promoting regional pollution control and precise management, specifically in areas with diverse pollution emission sources, such as urban–rural areas, or areas with diverse pollution pathways and dense river networks. Although the model needs further refining, this study provided a new perspective on source apportionment analysis for other researchers.

## Figures and Tables

**Table 1:** Descriptive statistics of heavy metals contents in soil of the study area (mg/kg).

Element	As	Cd	Cr	Cu	Ni	Pb	Zn	Ca	Mg
Minimum (mg/kg)	5.85	0.20	16.33	13.33	10.78	17.85	73.48	642.73	2662.55
Median (mg/kg)	26.72	0.54	81.89	27.42	27.8	40.35	131.97	1254.23	4444.38
Maximum (mg/kg)	83.81	2.48	219.75	65.46	71.95	95.85	305.63	5966.32	10,447.30
Mean (mg/kg)	26.47	0.70	87.07	29.37	28.41	43.94	133.78	1471.28	4600.78
Skewness	1.04	1.38	1.07	1.34	1.47	0.93	1.13	2.84	1.53
Kurtosis	2.21	2.69	1.42	2.57	5.19	0.84	3.86	10.84	3.85
SD ^a^	13.57	0.40	36.61	9.98	8.98	15.63	35.9	851.34	1323.58
CV% ^b^	51.27	57.21	42.05	33.99	31.59	35.57	26.83	57.86	28.77
Risk screening (mg/kg)	40.00	0.30	150.00	50.00	60.00	70.00	200.00	-	-
Background ^c^ (mg/kg)	12.80	0.14	150.00	25.00	27.80	30.00	84.20	1300.00	4000.00
Excess rate ^d^	16%	90%	8%	5%	2%	9%	5%	-	-

Note: ^a^ SD means standard deviation; ^b^ CV means coefficient of variation; ^c^ background values of heavy metals in Hunan Province; ^d^ percentage of points with soil heavy metal contents exceeding background values in Hunan Province.

**Table 2:** Contributions from sources and their ranges, calculated by the three models.

	Element	NMMI	CI	MMI	Soil Parent Material
Model 1	Cd	25%	19%	33%	23%
	As	17%	24%	42%	17%
	Pb	13%	24%	17%	46%
Model 2	Cd	28%	12%	44%	16%
	As	11%	14%	61%	14%
	Pb	1%	17%	21%	61%
Model 3	Cd	35%	20%	30%	15%
	As	10%	11%	58%	22%
	Pb	7%	24%	32%	37%
Model 4	Cd	54%	1%	8%	37%
	As	33%	47%	17%	3%
	Pb	17%	32%	41%	10%
Range (Models 1, 2, 3)	Cd	25–35%	12–20%	30–44%	16–23%
	As	10–17%	11–24%	42–58%	14–22%
	Pb	1–13%	17–24%	17–32%	37–61%

Note: NMMI: non-metal manufacturing factory; CI: chemical factory; MMI: metal manufacturing factory.

## Data Availability

Restrictions apply to the availability of these data.

## Supplementary Materials

The following supporting information can be downloaded at https://www.mdpi.com/article/10.3390/toxics12060382/s1, Table S1: Background values for different soil types.
