# Supplementary material for: A Modified Model for Quantitative Heavy Metal Source Apportionment and Pollution Pathway Identification
Source: Toxics. 2024 May 23;12(6):382. doi: 10.3390/toxics12060382 (PMC11209494; doi:10.3390/toxics12060382)
Supplement: Supplementary file 1 [file toxics-12-00382-s001.zip › toxics-3001726-supplementary.pdf]

Cover sheet for Supplementary Material for Publication

Maodi Wang [1], Pengyue Yu [1], Zhenglong Tong [1], Xingyuan Shao [1], Jianwei Peng [1], Yasir Hamid [2], Ying Huang [1,*]

[1] National Engineering Laboratory of High Efficient Use on Soil and Fertilizer, College of Resources, Hunan Agricultural University, Changsha 410128, China

[2] Ministry of Education (MOE) Key Lab of Environ. Remediation and Ecological Health, College of Environmental and Resources Science, Zhejiang University, Hangzhou 310058, China

Number of pages: 2

Number of tables: 1

Table content:

Table S1. Background values for different soil types.

Table S1. Background values for different soil types.

Soil types	**As**	**Cd**	**Cr**	**Cu**	**Ni**	**Pb**	**Zn**	**Ca**	**Mg**
Red earths	13.6	52.9	24.4	21.9	13.6	0.065	25.7	800	3400
Latosols	6.7	78.8	20	28.7	6.7	0.058	27.6	400	1600
Yellow earths	12.4	62	21.4	29.4	12.4	0.08	25.3	1200	4200
